# Supplementary material for: What is known from the existing literature about self-management of pessaries for pelvic organ prolapse? A scoping review protocol
Source: BMJ Open. 2022 Jan 11;12(1):e055587. doi: 10.1136/bmjopen-2021-055587 (PMC8753391; doi:10.1136/bmjopen-2021-055587)
Supplement: Author's manuscript [file bmjopen-2021-055587.draft_revisions.pdf]

BMJ Open

BMJ Open is committed to open peer review. As part of this commitment we make the peer review history of every article we publish publicly available.

When an article is published we post the peer reviewers' comments and the authors' responses online. We also post the versions of the paper that were used during peer review. These are the versions that the peer review comments apply to.

The versions of the paper that follow are the versions that were submitted during the peer review process. They are not the versions of record or the final published versions. They should not be cited or distributed as the published version of this manuscript.

BMJ Open is an open access journal and the full, final, typeset and author-corrected version of record of the manuscript is available on our site with no access controls, subscription charges or pay-per-view fees (http://bmjopen.bmj.com).

If you have any questions on BMJ Open's open peer review process please email info.bmjopen@bmj.com

BMJ Open

What is known from the existing literature about self-management of pessaries for pelvic organ prolapse? A scoping review protocol.

Journal:	*BMJ Open*
Manuscript ID	bmjopen-2021-055587
Article Type:	Protocol
Date Submitted by the Author:	19-Jul-2021
Complete List of Authors:	Dwyer, Lucy; Manchester Academic Health Science Centre; The University of Manchester<br>Dowding, Dawn; The University of Manchester, Division of Nursing, Midwifery and Social Work,<br>Kearney, R; Manchester Academic Health Science Centre, Saint Mary's Hospital, Manchester University NHS Foundation Trust, ; Manchester Academic Health Science Centre, Institute of Human Development, Faculty of Medical & Human Sciences
Keywords:	Urogynaecology < GYNAECOLOGY, Protocols & guidelines < HEALTH SERVICES ADMINISTRATION & MANAGEMENT, GYNAECOLOGY

SCHOLARONE™
Manuscripts

What is known from the existing literature about self-management of pessaries for pelvic organ prolapse? A scoping review protocol.

Dwyer. L[1,2], Dowding. D [2] and Kearney. R[1,3]

Affiliations:

[1] Saint Mary's Hospital, Manchester University NHS Foundation Trust, Manchester Academic Health Science Centre, Hathersage Road, Manchester M13 9WL, UK

[2]Division of Nursing, Midwifery and Social Work, School of Health Sciences, Faculty of Biology, Medicine and Health, University of Manchester, Oxford Road, Manchester, M13 9PL

[3] Institute of Human Development, Faculty of Medical & Human Sciences, University of Manchester, Oxford Road, Manchester, M13 9PL

Corresponding author: Lucy Dwyer

Lucy.dwyer@mft.nhs.uk 0161 276 6910

The Warrell Unit, Saint Mary's Hospital,

Manchester University NHS Foundation Trust

Hathersage Road

Manchester

M13 9WL

Dawn Dowding dawn.dowding@manchester.ac.uk

Rohna Kearney rohna.kearney@mft.nhs.uk

Abstract word count: 276

Main article word Count: 2359

Key words: Pessary, self-management, self-care, prolapse, scoping,

For peer review only

What is known from the existing literature about self-management of pessaries for pelvic organ prolapse? A scoping review protocol

Abstract

Introduction

Pelvic organ prolapse (POP) can be managed with a pessary [1]. However, regular follow-up required may deter women from pessary management due to the inconvenience of frequent appointments [2-4], as well as preventing pessary users from autonomous decision making. Pessary self-management, whereby the woman removes and inserts her own pessary may be a solution to these issues. However, there remains a number of uncertainties regarding the potential benefits and risks of pessary self-management [5]. This scoping review aims to map available evidence about the subject of pessary self-management for POP to identify knowledge gaps [6] providing the basis for future research.

Methods and analysis

The scoping review will be conducted utilising the Joanna Briggs Institute scoping review methodology [7] and reported in accordance with Preferred Reporting Items for Systematic reviews and Meta-Analyses extension for Scoping Reviews PRISMA-ScR guidelines [8]. A search of MEDLINE, CINAHL, EMBASE and PsycInfo will be undertaken to identify relevant articles which meet the eligibility criteria using the search terms 'pessary' and 'self-management' or 'self-care'. A hand search of the reference list of non-original research identified during the search but excluded, will be conducted for additional publications which meet the inclusion and exclusion criteria. Data relevant to the topic of pessary self-management will be extracted and critical appraisal of all included publications undertaken.

Ethics and dissemination

No ethical or Health Research Authority (HRA) approval is required to undertake the scoping review. However, it has been registered with The Open Science Framework (OSF) (DOI 10.17605/OSF.IO/DNGCP). The findings will inform future research exploring pessary self-management and be disseminated via both a presentation at a national conference and publications in peer reviewed journals.

For peer review only

Article Summary

Strengths and limitations of this study

- Understanding more about pessary self-management has been highlighted as a research priority by a group of pessary users and healthcare professionals.
- The scoping review will be undertaken following systematic and rigorous established guidelines to ensure transparency and reproducibility.
- Critical appraisal of the evidence will be undertaken to determine the weighting of included research findings, as well as identifying potential methodological strengths and limitations of the current evidence base.

For peer review only

Introduction

Pelvic organ prolapse (POP) is defined as the downward displacement of one or more of the pelvic organs including the uterus, vaginal compartments, bowel or bladder [9]. Common POP symptoms reported by women include seeing or feeling a vaginal bulge, a heaviness or a dragging sensation, difficulties voiding or defecating and sexual dysfunction, all of which can significantly negatively impact a woman's quality of life [9].

A pessary is a medical device which can be inserted into the vagina to provide mechanical support to the prolapsed organs. Pessary management offers women with prolapse comparable treatment outcomes to surgery in terms of reported symptoms and quality of life [1] and absence of bulge and no desire for further treatment [10]. This may be a particularly desirable option for women who have not completed their family, are unfit for surgical management or would simply prefer to avoid the risks that POP surgery entails [11]. There are a wide variety of pessaries available, offering a conservative, long-term management option to women. Pessary follow-up is required, tending to be 3-6 monthly [12]. At each appointment the pessary is removed, the vaginal tissues examined and either a new pessary, or the same pessary replaced after cleaning. The need for regular follow-up is often cited as a reason why women opt for surgical management of POP due to the inconvenience of frequent appointments [2, 3, 4]. Moreover, clinician management means women are denied autonomy in how and when to use their pessary. Furthermore, the cost of regular pessary follow-up appointments is not insignificant, with over 86,000 pessaries inserted annually in English NHS services alone [13].

A solution to these frequently cited issues with pessary care may be pessary self-management. This entails the woman removing and reinserting her own pessary as required or desired. There remains a number of uncertainties regarding the benefits and possible risks of pessary self-management [5]. A preliminary search in May 2021 identified a recent review of the published evidence related to pessary self-management, which highlighted current uncertainties within the subject area [5]. These include whether pessary self-management improves a woman's satisfaction, quality of life and therefore long-term use of a pessary; whether pessary self-management is safe and what follow-up requirements are for women self-managing their pessary [5]. However, the review was limited to searching PUBMED and had a focus of patient safety as indicated by the search terms utilised. Therefore, only five eligible studies were identified. A preliminary search of MEDLINE, the Cochrane Database of Systematic Reviews and JBI Evidence Synthesis was conducted and no further systematic or scoping reviews on the topic were identified as being currently underway.

It is acknowledged there is a lack of robust evidence regarding the risks and benefits of pessary self-management due to the small-scale, non-randomised nature of much of the evidence [5]. Whilst there is currently a lack of robust evidence regarding the benefits of self-management, there is qualitative and observational evidence from women using pessaries about the benefits pessary self-management offers to them, including flexibility in how and when they use the pessary [2, 14].

This scoping review aims to map available evidence about the subject of pessary self-management for POP to identify knowledge gaps [6] providing the basis for future research.

For peer review only - http://bmjopen.bmj.com/site/about/guidelines.xhtml

The following research question was formulated: What is known from the literature about pessary self-management for women with POP?

Methods and analysis

Types of Sources

The scoping review will be conducted utilising the Joanna Briggs Institute scoping review methodology [7] and reported in accordance with Preferred Reporting Items for Systematic reviews and Meta-Analyses extension for Scoping Reviews PRISMA-ScR guidelines (Figure 1) [8]. As advocated by PRISMA-ScR guidelines the review has been registered with The Open Science Framework (OSF) (DOI 10.17605/OSF.IO/DNGCP) and the protocol published to ensure transparency and reproducibility.

To be eligible for inclusion in the review, papers must include original research data regarding pessary self-management for women with POP. Pessary self-management and what it entails is poorly defined. However, for the purpose of this scoping review women who remove or insert their pessary independently will be classed as self-managing. There will be no time limit set as it is acknowledged pessary management of prolapse is a long-standing treatment option and therefore self-management of pessaries may also be. All identified studies will be included regardless of the methodology utilised to ensure the different aspects and perspectives of pessary self-management for POP will be explored. Non-original articles such as reviews will be excluded to avoid duplication of evidence. However, the reference list of all review articles identified in the search will be checked and all relevant original research cited will be included within this review.

The search aims to include publications from different methodological perspectives and to scope the available evidence related to pessary self-management for POP without presuming what the important outcomes may be. Therefore, rather than the PICO tool which is typically used for systematic reviews of quantitative evidence, the population, concept and context of the research question will be utilised to structure the search [15].

Population

The population in focus are women with pelvic organ prolapse who use a pessary. It is recognised the prevalence and severity of pelvic organ prolapse increases significantly with increasing age [16]. However, women of all ages develop prolapse, with 77% of 18-29-year-old women examined in a routine gynaecological clinic having stage one or two prolapse [16]. Therefore, no limits will be placed on the age of the study population. Pelvic organ prolapse is typically measured using the POPQ system [17], the measurements of which can be used to determine the stage of prolapse [17]. It is acknowledged the anatomical extent of a prolapse as measured by staging may not correlate with the severity of a woman's symptoms [18]. Therefore, it is advocated women be treated based upon the bothersomeness of their symptoms. It can thus be assumed women managing their prolapse symptoms with a pessary were sufficiently bothered to consent to treatment, and

For peer review only - http://bmjopen.bmj.com/site/about/guidelines.xhtml

the stage of their prolapse is of less significance than this. Therefore, the review will not exclude women based upon the stage of their prolapse.

Concept

The concept to be explored is specifically those who self-manage their pessary for pelvic organ prolapse. That included studies must relate to this type of pessary is an important distinction, as pessaries can be for either structural or medicinal purposes [19]. Pharmaceutical pessaries are solid tablets containing medicinal products for insertion into the vagina and are typically prescribed when a local effect is desired [19]. While there is limited evidence supporting the use of vaginal oestrogen as a treatment for pelvic organ prolapse [20], National Institute for Health and Care Excellence (NICE) guidelines suggest clinicians consider the use of local oestrogen cream or pessaries for women with symptomatic prolapse and vaginal atrophy [21]. Therefore, there is potential for articles with a focus of pharmaceutical pessaries for women with pelvic organ prolapse to be identified during the search. The term mechanical pessaries includes devices designed to manage symptoms of urinary incontinence [22], with some devices having a dual purpose for management of prolapse and urinary symptoms [22]. Studies will be excluded if the pessaries are solely for urinary incontinence, however studies with a sample population that includes women using dual purpose pessaries are eligible for inclusion. There is a broad range of pessaries available to manage pelvic organ prolapse and these are typically classed as either a support or space occupying pessary [17]. While the pessaries have different mechanisms of working, both have the same purpose of reducing the descent of, and symptoms associated with, prolapse. Therefore, studies including both types of pessaries will be included.

Context

Whether there are differences in the extent to which pessary self-management is offered to, or accepted by women, depending on the country they live in, healthcare provision, or the culture they belong to, is of particular interest to the authors. Therefore, there is no specific context to the question as the authors are interested in evidence related to pessary self-management for POP regardless of the setting where self-management was initiated, or within what country.

Articles will be excluded if they are not accessible in the English language due to feasibility issues, it is acknowledged this may limit the scope and generalisability of findings. Relevant conference abstracts will be included if the full data set has not subsequently been published. While this prevents the authors from appraising the full dataset and detailed description of the study processes, it enables the inclusion of recent research studies which have not yet been published as well as findings which may not be deemed sufficiently significant by the authors to publish.

The search strategy will aim to locate published studies related to pessary self-management. A search of MEDLINE, CINAHL, EMBASE and PsycInfo will be undertaken to identify articles

For peer review only

on the topic. The search terms which will be used for all databases are pessary and self-management or self-care. Searches of all databases will be undertaken in May 2021.

Inclusion criteria

- Original research
- Pessary for POP
- Published in English language
- Focuses on self-management of pessary for POP

Exclusion criteria

- Not relevant to subject area
- Not published in English language
- Not original research including case reports

Study/Source of Evidence selection

Following the search, all identified citations will be collated and uploaded and duplicates and non-original research publications removed. The abstracts will then be reviewed for relevance to the review question. In the instance of abstracts which do not explicitly refer to pessary self-management but are relevant to pessaries for prolapse, the full text will be reviewed to check for references to pessary self-management which may not have been deemed as sufficiently significant to include within the abstract. A sample of 20% of abstracts will be screened by an independent reviewer to ensure agreement with inclusion or exclusion decision. In the instance of disagreement regarding included or excluded studies not resolved through discussion, a third reviewer will be asked to make the final decision. Potentially relevant sources will be retrieved in full and assessed in detail against the inclusion criteria. Reasons for exclusion of sources of evidence that do not meet the inclusion criteria will be recorded and reported in the scoping review.

Data Extraction

Data will be extracted from papers included in the scoping review using a data extraction tool developed by the reviewers based upon scoping review guidance [7]. The extracted data will be entered into the tool electronically via Microsoft Excel. The data extraction form will include details of the author(s), year of publication, population studied, context, methodology utilised and key findings relevant to pessary self-management. A second reviewer will perform data extraction from a subset of 10% of included articles to ensure a standardised process. If there are discrepancies between both extractions, the potential reasons for this will be explored and the data extraction tool modified accordingly to reduce the likelihood for future discordance. Any amendments to the data extraction tool will be recorded including the reasons for this, for transparency. The final version of the data extraction tool will be included in the scoping review to ensure reproducibility.

For peer review only

Quality appraisal

Appraising the quality of included studies and the subsequent findings is not typically performed during a scoping review [15]. However, for the purpose of this review, quality will be assessed to determine the weighting of included research findings, as well as identifying potential methodological strengths and limitations of the current evidence base. As identified studies will be included regardless of the methodology utilised, it is important to identify a quality appraisal tool which can assess qualitative, quantitative and mixed methods research. Therefore, the updated Mixed Methods Appraisal Tool (MMAT) will be utilised [23]. The MMAT was initially developed in 2006 and has subsequently been revised to facilitate more efficient quality assessment, therefore the most recently developed 2018 MMAT will be used for this review [23]. The authors of the revised MMAT discourage reviewers from calculating a single overall score to determine the methodological quality of a paper as it prohibits readers from understanding the specific strengths and limitations of a study [23] Therefore, the quality appraisal of each included study will be presented in a tabular format (Figure 2), detailing the rating for each criterion assessed using the MMAT. The same subsample of studies randomly selected by the second reviewer for data extraction quality assurance, will be quality appraised by the second reviewer to ensure agreement in the assessment process. The 2018 version of the MMAT has improved content validity compared with previous versions, however the interrater reliability has yet to be established [24]. Therefore, ensuring there is concordance between both reviewer's assessment of quality will be an important step in the scoping review process.

Patient and public involvement

Members of the public and pessary users have not directly been involved with development of this protocol or review process. However, the need for research exploring pessary self-management was highlighted by The James Lind Alliance (JLA) Priority Setting Partnership for pessary and prolapse [25]. Several women with experience of pessaries participated in this partnership either as members of the steering group, by attending the consensus workshop or completing questionnaires. Understanding more about self-management was ranked third out of 20 priorities by the JLA Priority Setting Partnership. The topic of the scoping review has therefore previously been identified and prioritised by patients and members of the public.

Data Analysis and Presentation

The data will be analysed and presented in numerical and tabular format to describe the current evidence base, for example the extent of identified literature, the context of included research such as the country of origin and the nature of the research. This will provide a scope of the existing evidence related to pessary self-management as a key objective of this review. Furthermore, the key findings of included research will be analysed for emerging themes. A narrative description of the themes and a summary of the evidence related to this will be presented in text and numerical format as appropriate. The scoping review will be disseminated by publication in a peer reviewed journal and submitted for

For peer review only

presentation at a national conference. The findings will also inform subsequent exploratory work regarding pessary self-management.

For peer review only

Authors contributions

Lucy Dwyer devised the scoping review question, methodology and drafted this manuscript.

Rohna Kearney substantively contributed to the development of the scoping review question, methodology and revised and approved this manuscript.

Dawn Dowding substantively contributed to the development of the scoping review question, methodology and revised and approved this manuscript.

Competing interests statement

Lucy Dwyer and Rohna Kearney are co-applicants of the NIHR/HTA funded Treatment of Prolapse with Self-Care Pessary (TOPSY) study.

Funding

Lucy Dwyer, Clinical Doctoral Research Fellow, NIHR300519 is funded by Health Education England (HEE) / National Institute for Health Research (NIHR) for this research project. The views expressed in this publication are those of the author(s) and not necessarily those of the NIHR, NHS or the UK Department of Health and Social Care.

For peer review only

References

1. Lone F, Thakar R, Sultan AH. One-year prospective comparison of vaginal pessaries and surgery for pelvic organ prolapse using the validated ICIQ-VS and ICIQ-UI (SF) questionnaires. Int Urogynecol J. 2015 Sep;26(9):1305-12. doi: 10.1007/s00192-015-2686-9. Epub 2015 Apr 11. PMID: 25862241
2. Kearney R, Brown C. Self-management of vaginal pessaries for pelvic organ prolapse. BMJ Open Quality 2014;3:u206180.w2533. doi: 10.1136/bmjquality.u206180.w2533
3. Basu, M., Wise, B. and Duckett, J., 2011. A qualitative study of women's preferences for treatment of pelvic floor disorders. BJOG: An International Journal of Obstetrics & Gynaecology, 118(3), pp.338-344.
4. Nguyen, J. N. , Jones, C. R. & Krissovich, M. (2005). Pessary Treatment of Pelvic Relaxation. Journal of Wound, Ostomy and Continence Nursing, 32(4), 255–261.
5. Bugge C, Dembinsky M, Kearney R, Hagen S. Does self-management of vaginal pessaries improve care for women with pelvic organ prolapse? BMJ 2021; 372 :n310 doi:10.1136/bmj.n310
6. Munn, Z., Peters, M.D.J., Stern, C. *et al*, 2018. Systematic review or scoping review? Guidance for authors when choosing between a systematic or scoping review approach. BMC Med Res Methodol 18, 143 (2018).
7. Peters. MDJ, Godfrey. C, McInerney. P, Munn. Z, Tricco. AC, Khalil, H, 2020. Chapter 11: Scoping Reviews (2020 version). In: Aromataris E, Munn Z (Editors). JBI Manual for Evidence Synthesis,
8. Tricco AC, Lillie E, Zarin W, O'Brien KK, Colquhoun H, Levac D, *et al*. PRISMA Extension for Scoping Reviews (PRISMAScR): Checklist and Explanation. Ann Intern Med. 2018;169:467–473
9. Haylen, B.T., Maher, C.F., Barber, M.D. *et al*. An International Urogynecological Association (IUGA) / International Continence Society (ICS) joint report on the terminology for female pelvic organ prolapse (POP). Int Urogynecol J 27, 165–194 (2016). doi.org/10.1007/s00192-015-2932-1
10. Miceli, A., Dueñas-Diez, JL. Effectiveness of ring pessaries versus vaginal hysterectomy for advanced pelvic organ prolapse. A cohort study. Int Urogynecol J 30, 2161–2169 (2019). https://doi.org/10.1007/s00192-019-03919-8
11. Kapoor, D.S., Thakar, R., Sultan, A.H. *et al*. Conservative versus surgical management of prolapse: what dictates patient choice?. Int Urogynecol J 20, 1157 (2009). https://doi.org/10.1007/s00192-009-0930-x
12. Gorti M, Hudelist G, Simons A. Evaluation of vaginal pessary management: a UK-based survey. J Obstet Gynaecol. 2009 Feb;29(2):129-31. doi: 10.1080/01443610902719813. PMID: 19274547
13. Secondary Care Analytical Team, NHS Digital, 2020. Hospital Outpatient Activity 2019-20. Accessed online at digital.nhs.uk/data-and-information/publications/statistical/hospital-outpatient-activity on 10/05/21
14. Storey, S., Aston, M., Price, S., Irving, L. and Hemmens, E. (2009), Women's experiences with vaginal pessary use. Journal of Advanced Nursing, 65: 2350-2357. https://doi.org/10.1111/j.1365-2648.2009.05095.x

For peer review only

15. Peters, Micah D.J. Godfrey, Christina M. Khalil, Hanan; McInerney, Patricia Parker, Deborah; Soares, Cassia Baldini, 2015.s Guidance for conducting systematic scoping reviews, International Journal of Evidence-Based Healthcare: September 2015 - Volume 13 - Issue 3 - p 141-146 doi: 10.1097/XEB.0000000000000050
16. Swift. S, 2000. The distribution of pelvic organ support in a population of female subjects seen for routine gynecologic health care. American Journal of Obstetrics and Gynecology. Volume: 183 (2) 277-285
17. Dwyer. L and Kearney. R, 2021. Conservative management of pelvic organ prolapse, Obstetrics, Gynaecology & Reproductive Medicine, Volume 31 (2) 35-41
18. Gutman, R. E., Ford, D. E., Quiroz, L. H., Shippey, S. H., & Handa, V. L. (2008). Is there a pelvic organ prolapse threshold that predicts pelvic floor symptoms?. American journal of obstetrics and gynecology, 199(6), 683.e1–683.e6837.
19. Watson, J., Rees, J.A. and Smith, I. eds., 2014. Pharmaceutical practice. Elsevier Health Sciences
20. Ismail SI, Bain C, Hagen S. Oestrogens for treatment or prevention of pelvic organ prolapse in postmenopausal women. Cochrane Database of Systematic Reviews 2010, Issue 9. Art. No.: CD007063. DOI: 10.1002/14651858.CD007063.pub2. Accessed 08 June 2021.
21. National Institute for Health and Care Excellence, 2019. Urinary incontinence and pelvic organ prolapse in women: management. Accessed online at www.nice.org.uk/guidance/ng123 on 08/06/2021.
22. Amir-Khalkhali, B. and Farell, S.A., 2006. Selection of pessaries for urinary incontinence. In Pessaries in Clinical Practice (pp. 46-53). Springer, London.
23. Hong, Q.N., Fàbregues, S., Bartlett, G. *et al.* (10 more authors) (2018) The Mixed Methods Appraisal Tool (MMAT) version 2018 for information professionals and researchers. Education for Information, 34 (4). pp. 285-291. ISSN 0167-8329
24. Hong, Q. N., Pluye, P., Fàbregues, S., Bartlett, G., Boardman, F., Cargo, M., Dagenais, P., Gagnon, M. P., Griffiths, F., Nicolau, B., O'Cathain, A., Rousseau, M. C., & Vedel, I. (2019). Improving the content validity of the mixed methods appraisal tool: a modified e-Delphi study. Journal of clinical epidemiology, 111, 49–59.e1.
25. Lough K, Hagen S, McClurg D the JLA Pessary PSP Steering Group, *et al.* Shared research priorities for pessary use in women with prolapse: results from a James Lind Alliance Priority Setting Partnership. BMJ Open, April 2018, doi: 10.1136/bmjopen-2017-021276
26.

Figure legends

Figure 1- Preferred Reporting Items for Systematic reviews and Meta-Analyses extension for Scoping Reviews (PRISMA-ScR) Checklist

Figure 2- Example of presentation of Mixed Methods Appraisal Tool results

For peer review only

Preferred Reporting Items for Systematic reviews and Meta-Analyses extension for Scoping Reviews (PRISMA-ScR) Checklist

SECTION	ITEM	PRISMA-ScR CHECKLIST ITEM	REPORTED ON PAGE #
**TITLE**			
Title	1	Identify the report as a scoping review.	
**ABSTRACT**			
Structured summary	2	Provide a structured summary that includes (as applicable): background, objectives, eligibility criteria, sources of evidence, charting methods, results, and conclusions that relate to the review questions and objectives.	
**INTRODUCTION**			
Rationale	3	Describe the rationale for the review in the context of what is already known. Explain why the review questions/objectives lend themselves to a scoping review approach.	
Objectives	4	Provide an explicit statement of the questions and objectives being addressed with reference to their key elements (e.g., population or participants, concepts, and context) or other relevant key elements used to conceptualize the review questions and/or objectives.	
**METHODS**			
Protocol and registration	5	Indicate whether a review protocol exists; state if and where it can be accessed (e.g., a Web address); and if available, provide registration information, including the registration number.	
Eligibility criteria	6	Specify characteristics of the sources of evidence used as eligibility criteria (e.g., years considered, language, and publication status), and provide a rationale.	
Information sources*	7	Describe all information sources in the search (e.g., databases with dates of coverage and contact with authors to identify additional sources), as well as the date the most recent search was executed.	
Search	8	Present the full electronic search strategy for at least 1 database, including any limits used, such that it could be repeated.	
Selection of sources of evidence†	9	State the process for selecting sources of evidence (i.e., screening and eligibility) included in the scoping review.	
Data charting process‡	10	Describe the methods of charting data from the included sources of evidence (e.g., calibrated forms or forms that have been tested by the team before their use, and whether data charting was done independently or in duplicate) and any processes for obtaining and confirming data from investigators.	
Data items	11	List and define all variables for which data were sought and any assumptions and simplifications made.	
Critical appraisal of individual sources of evidence§	12	If done, provide a rationale for conducting a critical appraisal of included sources of evidence; describe the methods used and how this information was used in any data synthesis (if appropriate).	
Synthesis of results	13	Describe the methods of handling and summarizing the data that were charted.	

For peer review only

SECTION	ITEM	PRISMA-ScR CHECKLIST ITEM	REPORTED ON PAGE #
**RESULTS**			
Selection of sources of evidence	14	Give numbers of sources of evidence screened, assessed for eligibility, and included in the review, with reasons for exclusions at each stage, ideally using a flow diagram.	
Characteristics of sources of evidence	15	For each source of evidence, present characteristics for which data were charted and provide the citations.	
Critical appraisal within sources of evidence	16	If done, present data on critical appraisal of included sources of evidence (see item 12).	
Results of individual sources of evidence	17	For each included source of evidence, present the relevant data that were charted that relate to the review questions and objectives.	
Synthesis of results	18	Summarize and/or present the charting results as they relate to the review questions and objectives.	
**DISCUSSION**			
Summary of evidence	19	Summarize the main results (including an overview of concepts, themes, and types of evidence available), link to the review questions and objectives, and consider the relevance to key groups.	
Limitations	20	Discuss the limitations of the scoping review process.	
Conclusions	21	Provide a general interpretation of the results with respect to the review questions and objectives, as well as potential implications and/or next steps.	
**FUNDING**			
Funding	22	Describe sources of funding for the included sources of evidence, as well as sources of funding for the scoping review. Describe the role of the funders of the scoping review.	

JBI = Joanna Briggs Institute; PRISMA-ScR = Preferred Reporting Items for Systematic reviews and Meta-Analyses extension for Scoping Reviews.

* Where *sources of evidence* (see second footnote) are compiled from, such as bibliographic databases, social media platforms, and Web sites.

† A more inclusive/heterogeneous term used to account for the different types of evidence or data sources (e.g., quantitative and/or qualitative research, expert opinion, and policy documents) that may be eligible in a scoping review as opposed to only studies. This is not to be confused with *information sources* (see first footnote).

‡ The frameworks by Arksey and O'Malley (6) and Levac and colleagues (7) and the JBI guidance (4, 5) refer to the process of data extraction in a scoping review as data charting.

§ The process of systematically examining research evidence to assess its validity, results, and relevance before using it to inform a decision. This term is used for items 12 and 19 instead of "risk of bias" (which is more applicable to systematic reviews of interventions) to include and acknowledge the various sources of evidence that may be used in a scoping review (e.g., quantitative and/or qualitative research, expert opinion, and policy document).

From: Tricco AC, Lillie E, Zarin W, O'Brien KK, Colquhoun H, Levac D, et al. PRISMA Extension for Scoping Reviews (PRISMAScR): Checklist and Explanation. Ann Intern Med. 2018;169:467–473. doi: 10.7326/M18-0850.

St. Michael's
Inspired Care. Inspiring Science.

For peer review only - http://bmjopen.bmj.com/site/about/guidelines.xhtml

Figure 2

Studies	Criteria from the Mixed Methods Appraisal Tool																								
	1.1	1.2	1.3	1.4	1.5	2.1	2.2	2.3	2.4	2.5	3.1	3.2	3.3	3.4	3.5	4.1	4.2	4.3	4.4	4.5	5.1	5.2	5.3	5.4	5.5
Author, date	0	1	1	1	1											1	0	1	1	1	1	1	1	1	1
Author, date											0	1	1	1	1										
Author, date						1	1	1	0	1															

http://mixedmethodsappraisaltoolpublic.pbworks.com/w/file/fetch/140056890/Reporting%20the%20results%20of%20the%20MMAT.pdf

For peer review only

BMJ Open

What is known from the existing literature about self-management of pessaries for pelvic organ prolapse? A scoping review protocol.

Journal:	*BMJ Open*
Manuscript ID	bmjopen-2021-055587.R1
Article Type:	Protocol
Date Submitted by the Author:	01-Nov-2021
Complete List of Authors:	Dwyer, Lucy; Manchester Academic Health Science Centre; The University of Manchester<br>Dowding, Dawn; The University of Manchester, Division of Nursing, Midwifery and Social Work,<br>Kearney, R; Manchester Academic Health Science Centre, Saint Mary's Hospital, Manchester University NHS Foundation Trust, ; Manchester Academic Health Science Centre, Institute of Human Development, Faculty of Medical & Human Sciences
**Primary Subject Heading**:	Obstetrics and gynaecology
Secondary Subject Heading:	Nursing
Keywords:	Urogynaecology < GYNAECOLOGY, Protocols & guidelines < HEALTH SERVICES ADMINISTRATION & MANAGEMENT, GYNAECOLOGY

SCHOLARONE™
Manuscripts

What is known from the existing literature about self-management of pessaries for pelvic organ prolapse? A scoping review protocol.

Dwyer. L[1,2], Dowding. D [2] and Kearney. R[1,3]

Affiliations:

[1] Saint Mary's Hospital, Manchester University NHS Foundation Trust, Manchester Academic Health Science Centre, Hathersage Road, Manchester M13 9WL, UK

[2]Division of Nursing, Midwifery and Social Work, School of Health Sciences, Faculty of Biology, Medicine and Health, University of Manchester, Oxford Road, Manchester, M13 9PL

[3] Institute of Human Development, Faculty of Medical & Human Sciences, University of Manchester, Oxford Road, Manchester, M13 9PL

Corresponding author: Lucy Dwyer

Lucy.dwyer@mft.nhs.uk 0161 276 6910

The Warrell Unit, Saint Mary's Hospital,

Manchester University NHS Foundation Trust

Hathersage Road

Manchester

M13 9WL

Dawn Dowding dawn.dowding@manchester.ac.uk

Rohna Kearney rohna.kearney@mft.nhs.uk

Abstract word count: 276

Main article word Count: 2416

Key words: Pessary, self-management, self-care, prolapse, scoping,

For peer review only

What is known from the existing literature about self-management of pessaries for pelvic organ prolapse? A scoping review protocol

Abstract

Introduction

Pelvic organ prolapse (POP) can be managed with a pessary [1]. However, regular follow-up required may deter women from pessary management due to the inconvenience of frequent appointments [2-4], as well as preventing pessary users from autonomous decision making. Pessary self-management, whereby the woman removes and inserts her own pessary may be a solution to these issues. However, there remains a number of uncertainties regarding the potential benefits and risks of pessary self-management [5]. This scoping review aims to map available evidence about the subject of pessary self-management for POP to identify knowledge gaps [6] providing the basis for future research.

Methods and analysis

The scoping review will be conducted utilising the Joanna Briggs Institute scoping review methodology [7] and reported in accordance with Preferred Reporting Items for Systematic reviews and Meta-Analyses extension for Scoping Reviews PRISMA-ScR guidelines [8]. A search of MEDLINE, CINAHL, EMBASE and PsycInfo will be undertaken to identify relevant articles which meet the eligibility criteria using the search terms 'pessary' and 'self-management' or 'self-care'. A hand search of the reference list of non-original research identified during the search but excluded, will be conducted for additional publications which meet the inclusion and exclusion criteria. Data relevant to the topic of pessary self-management will be extracted and critical appraisal of all included publications undertaken.

Ethics and dissemination

No ethical or Health Research Authority (HRA) approval is required to undertake the scoping review. However, it has been registered with The Open Science Framework (OSF) (DOI 10.17605/OSF.IO/DNGCP). The findings will inform future research exploring pessary self-management and be disseminated via both a presentation at a national conference and publications in peer reviewed journals.

For peer review only

Article Summary

Strengths and limitations of this study

- The scoping review will be undertaken following systematic and rigorous established guidelines to ensure transparency and reproducibility.
- Critical appraisal of the evidence will be undertaken to determine the weighting of included research findings, as well as identifying potential methodological strengths and limitations of the current evidence base.
- The identification and synthesis of data will be limited to published articles found on the MEDLINE, CINAHL, EMBASE and PsycInfo databases and a hand search of reference lists.

For peer review only

Introduction

Pelvic organ prolapse (POP) is defined as the downward displacement of one or more of the pelvic organs including the uterus, vaginal compartments, bowel or bladder [9]. Common POP symptoms reported by women include seeing or feeling a vaginal bulge, a heaviness or a dragging sensation, difficulties voiding or defecating and sexual dysfunction, all of which can significantly negatively impact a woman's quality of life [9].

A pessary is a medical device which can be inserted into the vagina to provide mechanical support to the prolapsed organs. Pessary management offers women with prolapse comparable treatment outcomes to surgery in terms of reported symptoms and quality of life [1] and absence of bulge and no desire for further treatment [10]. This may be a particularly desirable option for women who have not completed their family, are unfit for surgical management or would simply prefer to avoid the risks that POP surgery entails [11]. There are a wide variety of pessaries available, offering a conservative, long-term management option to women. Pessary follow-up is required, tending to be 3-6 monthly [12]. At each appointment the pessary is removed, the vaginal tissues examined and either a new pessary, or the same pessary replaced after cleaning. The need for regular follow-up is often cited as a reason why women opt for surgical management of POP due to the inconvenience of frequent appointments [2, 3, 4]. Moreover, clinician management means women are denied autonomy in how and when to use their pessary. Furthermore, the cost of regular pessary follow-up appointments is not insignificant, with over 86,000 pessaries inserted annually in English NHS services alone [13].

A solution to these frequently cited issues with pessary care may be pessary self-management. This entails the woman removing and reinserting her own pessary as required or desired. There remains a number of uncertainties regarding the benefits and possible risks of pessary self-management [5]. A preliminary search in May 2021 identified a recent review of the published evidence related to pessary self-management, which highlighted current uncertainties within the subject area [5]. These include whether pessary self-management improves a woman's satisfaction, quality of life and therefore long-term use of a pessary; whether pessary self-management is safe and what follow-up requirements are for women self-managing their pessary [5]. However, the review was limited to searching PUBMED and had a focus of patient safety as indicated by the search terms utilised. Therefore, only five eligible studies were identified. A preliminary search of MEDLINE, the Cochrane Database of Systematic Reviews and JBI Evidence Synthesis was conducted and no further systematic or scoping reviews on the topic were identified as being currently underway.

It is acknowledged there is a lack of robust evidence regarding the risks and benefits of pessary self-management due to the small-scale, non-randomised nature of much of the evidence [5]. Whilst there is currently a lack of robust evidence regarding the benefits of self-management, there is qualitative and observational evidence from women using pessaries about the benefits pessary self-management offers to them, including flexibility in how and when they use the pessary [2, 14].

This scoping review aims to map available evidence about the subject of pessary self-management for POP to identify knowledge gaps [6] providing the basis for future research.

For peer review only

The following research question was formulated: What is known from the literature about pessary self-management for women with POP?

Methods and analysis

Types of Sources

The scoping review will be conducted utilising the Joanna Briggs Institute scoping review methodology [7] and reported in accordance with Preferred Reporting Items for Systematic reviews and Meta-Analyses extension for Scoping Reviews PRISMA-ScR guidelines (Figure 1) [8]. As advocated by PRISMA-ScR guidelines the review has been registered with The Open Science Framework (OSF) (DOI 10.17605/OSF.IO/DNGCP) and the protocol published to ensure transparency and reproducibility.

To be eligible for inclusion in the review, papers must include original research data regarding pessary self-management for women with POP. Pessary self-management and what it entails is poorly defined. However, for the purpose of this scoping review women who remove or insert their pessary independently will be classed as self-managing. There will be no time limit set as it is acknowledged pessary management of prolapse is a long-standing treatment option and therefore self-management of pessaries may also be. All identified studies will be included regardless of the methodology utilised to ensure the different aspects and perspectives of pessary self-management for POP will be explored. Non-original articles such as reviews will be excluded to avoid duplication of evidence. However, the reference list of all review articles identified in the search will be checked and all relevant original research cited will be included within this review.

The search aims to include publications from different methodological perspectives and to scope the available evidence related to pessary self-management for POP without presuming what the important outcomes may be. Therefore, rather than the PICO tool which is typically used for systematic reviews of quantitative evidence, the population, concept and context of the research question will be utilised to structure the search [15].

Population

The population in focus are women with pelvic organ prolapse who use a pessary. It is recognised the prevalence and severity of pelvic organ prolapse increases significantly with increasing age [16]. However, women of all ages develop prolapse, with 77% of 18-29-year-old women examined in a routine gynaecological clinic having stage one or two prolapse [16]. Therefore, no limits will be placed on the age of the study population. Pelvic organ prolapse is typically measured using the POPQ system [17], the measurements of which can be used to determine the stage of prolapse [17]. It is acknowledged the anatomical extent of a prolapse as measured by staging may not correlate with the severity of a woman's symptoms [18]. Therefore, it is advocated women be treated based upon the bothersomeness of their symptoms. It can thus be assumed women managing their prolapse symptoms with a pessary were sufficiently bothered to consent to treatment, and

For peer review only

the stage of their prolapse is of less significance than this. Therefore, the review will not exclude women based upon the stage of their prolapse.

Concept

The concept to be explored is specifically those who self-manage their pessary for pelvic organ prolapse. That included studies must relate to this type of pessary is an important distinction, as pessaries can be for either structural or medicinal purposes [19]. Pharmaceutical pessaries are solid tablets containing medicinal products for insertion into the vagina and are typically prescribed when a local effect is desired [19]. While there is limited evidence supporting the use of vaginal oestrogen as a treatment for pelvic organ prolapse [20], National Institute for Health and Care Excellence (NICE) guidelines suggest clinicians consider the use of local oestrogen cream or pessaries for women with symptomatic prolapse and vaginal atrophy [21]. Therefore, there is potential for articles with a focus of pharmaceutical pessaries for women with pelvic organ prolapse to be identified during the search. The term mechanical pessaries includes devices designed to manage symptoms of urinary incontinence [22], with some devices having a dual purpose for management of prolapse and urinary symptoms [22]. Studies will be excluded if the pessaries are solely for urinary incontinence, however studies with a sample population that includes women using dual purpose pessaries are eligible for inclusion. There is a broad range of pessaries available to manage pelvic organ prolapse and these are typically classed as either a support or space occupying pessary [17]. While the pessaries have different mechanisms of working, both have the same purpose of reducing the descent of, and symptoms associated with, prolapse. Therefore, studies including both types of pessaries will be included.

Context

Whether there are differences in the extent to which pessary self-management is offered to, or accepted by women, depending on the country they live in, healthcare provision, or the culture they belong to, is of particular interest to the authors. Therefore, there is no specific context to the question as the authors are interested in evidence related to pessary self-management for POP regardless of the setting where self-management was initiated, or within what country.

Articles will be excluded if they are not accessible in the English language due to feasibility issues, it is acknowledged this may limit the scope and generalisability of findings. Relevant conference abstracts will be included if the full data set has not subsequently been published. While this prevents the authors from appraising the full dataset and detailed description of the study processes, it enables the inclusion of recent research studies which have not yet been published as well as findings which may not be deemed sufficiently significant by the authors to publish.

The search strategy will aim to locate published studies related to pessary self-management. A search of MEDLINE, CINAHL, EMBASE and PsycInfo will be undertaken to identify articles on the topic. The search terms which will be used for all databases are pessary and self-

For peer review only

management or self-care. Searches of all databases will be undertaken between 5th and 7th of May 2021. Hand searches will be undertaken throughout May 2021. Data extraction, critical appraisal and synthesis of the results will be undertaken following this.

Inclusion criteria

- Original research
- Pessary for POP
- Published in English language
- Focuses on self-management of pessary for POP

Exclusion criteria

- Not relevant to subject area
- Not published in English language
- Not original research including case reports

For peer review only

Study/Source of Evidence selection

Following the search, all identified citations will be collated and uploaded and duplicates and non-original research publications removed. The abstracts will then be reviewed for relevance to the review question. In the instance of abstracts which do not explicitly refer to pessary self-management but are relevant to pessaries for prolapse, the full text will be reviewed to check for references to pessary self-management which may not have been deemed as sufficiently significant to include within the abstract. All data identified which is relevant to pessary self-management will be extracted regardless of the overall aim of the publication, the care setting and the level of focus upon pessary self-management. A sample of 20% of abstracts will be screened by an independent reviewer to ensure agreement with inclusion or exclusion decision. In the instance of disagreement regarding included or excluded studies not resolved through discussion, a third reviewer will be asked to make the final decision. Potentially relevant sources will be retrieved in full and assessed in detail against the inclusion criteria. Reasons for exclusion of sources of evidence that do not meet the inclusion criteria will be recorded and reported in the scoping review.

Data Extraction

Data will be extracted from papers included in the scoping review using a data extraction tool developed by the reviewers based upon scoping review guidance [7]. The extracted data will be entered into the tool electronically via Microsoft Excel. The data extraction form will include details of the author(s), year of publication, population studied, context, methodology utilised and key findings relevant to pessary self-management. A second reviewer will perform data extraction from a subset of 10% of included articles to ensure a standardised process. If there are discrepancies between both extractions, the potential reasons for this will be explored and the data extraction tool modified accordingly to reduce

the likelihood for future discordance. Any amendments to the data extraction tool will be recorded including the reasons for this, for transparency. The final version of the data extraction tool will be included in the scoping review to ensure reproducibility.

Quality appraisal

Appraising the quality of included studies and the subsequent findings is not typically performed during a scoping review [15]. However, for the purpose of this review, quality will be assessed to determine the weighting of included research findings, as well as identifying potential methodological strengths and limitations of the current evidence base. As identified studies will be included regardless of the methodology utilised, it is important to identify a quality appraisal tool which can assess qualitative, quantitative and mixed methods research. Therefore, the updated Mixed Methods Appraisal Tool (MMAT) will be utilised [23]. The MMAT was initially developed in 2006 and has subsequently been revised to facilitate more efficient quality assessment, therefore the most recently developed 2018 MMAT will be used for this review [23]. The authors of the revised MMAT discourage reviewers from calculating a single overall score to determine the methodological quality of a paper as it prohibits readers from understanding the specific strengths and limitations of a study [23] Therefore, the quality appraisal of each included study will be presented in a tabular format (Figure 2), detailing the rating for each criterion assessed using the MMAT. The same subsample of studies randomly selected by the second reviewer for data extraction quality assurance, will be quality appraised by the second reviewer to ensure agreement in the assessment process. The 2018 version of the MMAT has improved content validity compared with previous versions, however the interrater reliability has yet to be established [24]. Therefore, ensuring there is concordance between both reviewer's assessment of quality will be an important step in the scoping review process.

Patient and public involvement

Members of the public and pessary users have not directly been involved with development of this protocol or review process. However, the need for research exploring pessary self-management was highlighted by The James Lind Alliance (JLA) Priority Setting Partnership for pessary and prolapse [25]. Several women with experience of pessaries participated in this partnership either as members of the steering group, by attending the consensus workshop or completing questionnaires. Understanding more about self-management was ranked third out of 20 priorities by the JLA Priority Setting Partnership. The topic of the scoping review has therefore previously been identified and prioritised by patients and members of the public.

Data Analysis and Presentation

The data will be analysed and presented in numerical and tabular format to describe the current evidence base, for example the extent of identified literature, the context of included research such as the country of origin and the nature of the research. This will provide a scope of the existing evidence related to pessary self-management as a key

For peer review only - http://bmjopen.bmj.com/site/about/guidelines.xhtml

objective of this review. Furthermore, the key findings of included research will be analysed for emerging themes. A narrative description of the themes and a summary of the evidence related to this will be presented in text and numerical format as appropriate. The scoping review will be disseminated by publication in a peer reviewed journal and submitted for presentation at a national conference. The findings will also inform subsequent exploratory work regarding pessary self-management.

For peer review only

Authors contributions

Lucy Dwyer devised the scoping review question, methodology and drafted this manuscript.

Rohna Kearney substantively contributed to the development of the scoping review question, methodology and revised and approved this manuscript.

Dawn Dowding substantively contributed to the development of the scoping review question, methodology and revised and approved this manuscript.

Competing interests statement

Lucy Dwyer and Rohna Kearney are co-applicants of the NIHR/HTA funded Treatment of Prolapse with Self-Care Pessary (TOPSY) study.

Funding

Lucy Dwyer, Clinical Doctoral Research Fellow, NIHR300519 is funded by Health Education England (HEE) / National Institute for Health Research (NIHR) for this research project. The views expressed in this publication are those of the author(s) and not necessarily those of the NIHR, NHS or the UK Department of Health and Social Care.

For peer review only

References

1. Lone F, Thakar R, Sultan AH. One-year prospective comparison of vaginal pessaries and surgery for pelvic organ prolapse using the validated ICIQ-VS and ICIQ-UI (SF) questionnaires. Int Urogynecol J. 2015 Sep;26(9):1305-12. doi: 10.1007/s00192-015-2686-9. Epub 2015 Apr 11. PMID: 25862241
2. Kearney R, Brown C. Self-management of vaginal pessaries for pelvic organ prolapse. BMJ Open Quality 2014;3:u206180.w2533. doi: 10.1136/bmjquality.u206180.w2533
3. Basu, M., Wise, B. and Duckett, J., 2011. A qualitative study of women's preferences for treatment of pelvic floor disorders. BJOG: An International Journal of Obstetrics & Gynaecology, 118(3), pp.338-344.
4. Nguyen, J. N. , Jones, C. R. & Krissovich, M. (2005). Pessary Treatment of Pelvic Relaxation. Journal of Wound, Ostomy and Continence Nursing, 32(4), 255–261.
5. Bugge C, Dembinsky M, Kearney R, Hagen S. Does self-management of vaginal pessaries improve care for women with pelvic organ prolapse? BMJ 2021; 372 :n310 doi:10.1136/bmj.n310
6. Munn, Z., Peters, M.D.J., Stern, C. *et al*, 2018. Systematic review or scoping review? Guidance for authors when choosing between a systematic or scoping review approach. BMC Med Res Methodol 18, 143 (2018).
7. Peters. MDJ, Godfrey. C, McInerney. P, Munn. Z, Tricco. AC, Khalil, H, 2020. Chapter 11: Scoping Reviews (2020 version). In: Aromataris E, Munn Z (Editors). JBI Manual for Evidence Synthesis,
8. Tricco AC, Lillie E, Zarin W, O'Brien KK, Colquhoun H, Levac D, *et al*. PRISMA Extension for Scoping Reviews (PRISMAScR): Checklist and Explanation. Ann Intern Med. 2018;169:467–473
9. Haylen, B.T., Maher, C.F., Barber, M.D. *et al*. An International Urogynecological Association (IUGA) / International Continence Society (ICS) joint report on the terminology for female pelvic organ prolapse (POP). Int Urogynecol J 27, 165–194 (2016). doi.org/10.1007/s00192-015-2932-1
10. Miceli, A., Dueñas-Diez, JL. Effectiveness of ring pessaries versus vaginal hysterectomy for advanced pelvic organ prolapse. A cohort study. Int Urogynecol J 30, 2161–2169 (2019). https://doi.org/10.1007/s00192-019-03919-8
11. Kapoor, D.S., Thakar, R., Sultan, A.H. *et al*. Conservative versus surgical management of prolapse: what dictates patient choice?. Int Urogynecol J 20, 1157 (2009). https://doi.org/10.1007/s00192-009-0930-x
12. Gorti M, Hudelist G, Simons A. Evaluation of vaginal pessary management: a UK-based survey. J Obstet Gynaecol. 2009 Feb;29(2):129-31. doi: 10.1080/01443610902719813. PMID: 19274547
13. Secondary Care Analytical Team, NHS Digital, 2020. Hospital Outpatient Activity 2019-20. Accessed online at digital.nhs.uk/data-and-information/publications/statistical/hospital-outpatient-activity on 10/05/21
14. Storey, S., Aston, M., Price, S., Irving, L. and Hemmens, E. (2009), Women's experiences with vaginal pessary use. Journal of Advanced Nursing, 65: 2350-2357. https://doi.org/10.1111/j.1365-2648.2009.05095.x

For peer review only

15. Peters, Micah D.J. Godfrey, Christina M. Khalil, Hanan; McInerney, Patricia Parker, Deborah; Soares, Cassia Baldini, 2015.s Guidance for conducting systematic scoping reviews, International Journal of Evidence-Based Healthcare: September 2015 - Volume 13 - Issue 3 - p 141-146 doi: 10.1097/XEB.0000000000000050
16. Swift. S, 2000. The distribution of pelvic organ support in a population of female subjects seen for routine gynecologic health care. American Journal of Obstetrics and Gynecology. Volume: 183 (2) 277-285
17. Dwyer. L and Kearney. R, 2021. Conservative management of pelvic organ prolapse, Obstetrics, Gynaecology & Reproductive Medicine, Volume 31 (2) 35-41
18. Gutman, R. E., Ford, D. E., Quiroz, L. H., Shippey, S. H., & Handa, V. L. (2008). Is there a pelvic organ prolapse threshold that predicts pelvic floor symptoms?. American journal of obstetrics and gynecology, 199(6), 683.e1–683.e6837.
19. Watson, J., Rees, J.A. and Smith, I. eds., 2014. Pharmaceutical practice. Elsevier Health Sciences
20. Ismail SI, Bain C, Hagen S. Oestrogens for treatment or prevention of pelvic organ prolapse in postmenopausal women. Cochrane Database of Systematic Reviews 2010, Issue 9. Art. No.: CD007063. DOI: 10.1002/14651858.CD007063.pub2. Accessed 08 June 2021.
21. National Institute for Health and Care Excellence, 2019. Urinary incontinence and pelvic organ prolapse in women: management. Accessed online at www.nice.org.uk/guidance/ng123 on 08/06/2021.
22. Amir-Khalkhali, B. and Farell, S.A., 2006. Selection of pessaries for urinary incontinence. In Pessaries in Clinical Practice (pp. 46-53). Springer, London.
23. Hong, Q.N., Fàbregues, S., Bartlett, G. *et al.* (10 more authors) (2018) The Mixed Methods Appraisal Tool (MMAT) version 2018 for information professionals and researchers. Education for Information, 34 (4). pp. 285-291. ISSN 0167-8329
24. Hong, Q. N., Pluye, P., Fàbregues, S., Bartlett, G., Boardman, F., Cargo, M., Dagenais, P., Gagnon, M. P., Griffiths, F., Nicolau, B., O'Cathain, A., Rousseau, M. C., & Vedel, I. (2019). Improving the content validity of the mixed methods appraisal tool: a modified e-Delphi study. Journal of clinical epidemiology, 111, 49–59.e1.
25. Lough K, Hagen S, McClurg D the JLA Pessary PSP Steering Group, *et al.* Shared research priorities for pessary use in women with prolapse: results from a James Lind Alliance Priority Setting Partnership. BMJ Open, April 2018, doi: 10.1136/bmjopen-2017-021276

For peer review only

Figure legends

Figure 1- Preferred Reporting Items for Systematic reviews and Meta-Analyses extension for Scoping Reviews (PRISMA-ScR) Checklist

Figure 2- Example of presentation of Mixed Methods Appraisal Tool results

For peer review only

Preferred Reporting Items for Systematic reviews and Meta-Analyses extension for Scoping Reviews (PRISMA-ScR) Checklist

SECTION	ITEM	PRISMA-ScR CHECKLIST ITEM	REPORTED ON PAGE #
**TITLE**			
Title	1	Identify the report as a scoping review.	1
**ABSTRACT**			
Structured summary	2	Provide a structured summary that includes (as applicable): background, objectives, eligibility criteria, sources of evidence, charting methods, results, and conclusions that relate to the review questions and objectives.	2
**INTRODUCTION**			
Rationale	3	Describe the rationale for the review in the context of what is already known. Explain why the review questions/objectives lend themselves to a scoping review approach.	4
Objectives	4	Provide an explicit statement of the questions and objectives being addressed with reference to their key elements (e.g., population or participants, concepts, and context) or other relevant key elements used to conceptualize the review questions and/or objectives.	4
**METHODS**			
Protocol and registration	5	Indicate whether a review protocol exists; state if and where it can be accessed (e.g., a Web address); and if available, provide registration information, including the registration number.	5
Eligibility criteria	6	Specify characteristics of the sources of evidence used as eligibility criteria (e.g., years considered, language, and publication status), and provide a rationale.	5 and 7
Information sources*	7	Describe all information sources in the search (e.g., databases with dates of coverage and contact with authors to identify additional sources), as well as the date the most recent search was executed.	5
Search	8	Present the full electronic search strategy for at least 1 database, including any limits used, such that it could be repeated.	Included
Selection of sources of evidence†	9	State the process for selecting sources of evidence (i.e., screening and eligibility) included in the scoping review.	7
Data charting process‡	10	Describe the methods of charting data from the included sources of evidence (e.g., calibrated forms or forms that have been tested by the team before their use, and whether data charting was done independently or in duplicate) and any processes for obtaining and confirming data from investigators.	7
Data items	11	List and define all variables for which data were sought and any assumptions and simplifications made.	5-7
Critical appraisal of individual sources of evidence§	12	If done, provide a rationale for conducting a critical appraisal of included sources of evidence; describe the methods used and how this information was used in any data synthesis (if appropriate).	8

St. Michael's
Inspired Care.
Inspiring Science.

For peer review only - http://bmjopen.bmj.com/site/about/guidelines.xhtml

SECTION	ITEM	PRISMA-ScR CHECKLIST ITEM	REPORTED ON PAGE #
Synthesis of results	13	Describe the methods of handling and summarizing the data that were charted.	8
**RESULTS**			
Selection of sources of evidence	14	Give numbers of sources of evidence screened, assessed for eligibility, and included in the review, with reasons for exclusions at each stage, ideally using a flow diagram.	n/a-to be published with results
Characteristics of sources of evidence	15	For each source of evidence, present characteristics for which data were charted and provide the citations.	n/a-to be published with results
Critical appraisal within sources of evidence	16	If done, present data on critical appraisal of included sources of evidence (see item 12).	n/a-to be published with results
Results of individual sources of evidence	17	For each included source of evidence, present the relevant data that were charted that relate to the review questions and objectives.	n/a-to be published with results
Synthesis of results	18	Summarize and/or present the charting results as they relate to the review questions and objectives.	n/a-to be published with results
**DISCUSSION**			
Summary of evidence	19	Summarize the main results (including an overview of concepts, themes, and types of evidence available), link to the review questions and objectives, and consider the relevance to key groups.	n/a-to be published with results
Limitations	20	Discuss the limitations of the scoping review process.	n/a-to be published with results
Conclusions	21	Provide a general interpretation of the results with respect to the review questions and objectives, as well as potential implications and/or next steps.	n/a-to be published with results
**FUNDING**			
Funding	22	Describe sources of funding for the included sources of evidence, as well as sources of funding for the scoping review. Describe the role of the funders of the scoping review.	10

JBI = Joanna Briggs Institute; PRISMA-ScR = Preferred Reporting Items for Systematic reviews and Meta-Analyses extension for Scoping Reviews.
* Where *sources of evidence* (see second footnote) are compiled from, such as bibliographic databases, social media platforms, and Web sites.
† A more inclusive/heterogeneous term used to account for the different types of evidence or data sources (e.g., quantitative and/or qualitative research, expert opinion, and policy documents) that may be eligible in a scoping review as opposed to only studies. This is not to be confused with *information sources* (see first footnote).
‡ The frameworks by Arksey and O'Malley (6) and Levac and colleagues (7) and the JBI guidance (4, 5) refer to the process of data extraction in a scoping review as data charting.
§ The process of systematically examining research evidence to assess its validity, results, and relevance before using it to inform a decision. This term is used for items 12 and 19 instead of "risk of bias" (which is more applicable to systematic reviews of interventions) to include and acknowledge the various sources of evidence that may be used in a scoping review (e.g., quantitative and/or qualitative research, expert opinion, and policy document).

From: Tricco AC, Lillie E, Zarin W, O'Brien KK, Colquhoun H, Levac D, et al. PRISMA Extension for Scoping Reviews (PRISMAScR): Checklist and Explanation. Ann Intern Med. 2018;169:467–473. doi: 10.7326/M18-0850.

For peer review only - http://bmjopen.bmj.com/site/about/guidelines.xhtml

Figure 2

**Studies**	**Criteria from the Mixed Methods Appraisal Tool**																								
	1.1	1.2	1.3	1.4	1.5	2.1	2.2	2.3	2.4	2.5	3.1	3.2	3.3	3.4	3.5	4.1	4.2	4.3	4.4	4.5	5.1	5.2	5.3	5.4	5.5
Author, date	0	1	1	1	1											1	0	1	1	1	1	1	1	1	1
Author, date											0	1	1	1	1										
Author, date						1	1	1	0	1															

For peer review only

http://mixedmethodsappraisaltoolpublic.pbworks.com/w/file/fetch/140056890/Reporting%20the%20results%20of%20the%20MMAT.pdf

For peer review only - http://bmjopen.bmj.com/site/about/guidelines.xhtml

BMJ Open

What is known from the existing literature about self-management of pessaries for pelvic organ prolapse? A scoping review protocol.

Journal:	*BMJ Open*
Manuscript ID	bmjopen-2021-055587.R2
Article Type:	Protocol
Date Submitted by the Author:	07-Dec-2021

Complete List of Authors:	Dwyer, Lucy; Manchester Academic Health Science Centre; The University of Manchester<br>Dowding, Dawn; The University of Manchester, Division of Nursing, Midwifery and Social Work,<br>Kearney, R; Manchester Academic Health Science Centre, Saint Mary’s Hospital, Manchester University NHS Foundation Trust, ; Manchester Academic Health Science Centre, Institute of Human Development, Faculty of Medical & Human Sciences

**Primary Subject Heading**:	Obstetrics and gynaecology
Secondary Subject Heading:	Nursing
Keywords:	Urogynaecology < GYNAECOLOGY, Protocols & guidelines < HEALTH SERVICES ADMINISTRATION & MANAGEMENT, GYNAECOLOGY

SCHOLARONE™
Manuscripts

What is known from the existing literature about self-management of pessaries for pelvic organ prolapse? A scoping review protocol.

Dwyer. L[1,2], Dowding. D [2] and Kearney. R[1,3]

Affiliations:

[1] Saint Mary's Hospital, Manchester University NHS Foundation Trust, Manchester Academic Health Science Centre, Hathersage Road, Manchester M13 9WL, UK

[2]Division of Nursing, Midwifery and Social Work, School of Health Sciences, Faculty of Biology, Medicine and Health, University of Manchester, Oxford Road, Manchester, M13 9PL

[3] Institute of Human Development, Faculty of Medical & Human Sciences, University of Manchester, Oxford Road, Manchester, M13 9PL

Corresponding author: Lucy Dwyer

Lucy.dwyer@mft.nhs.uk 0161 276 6910

The Warrell Unit, Saint Mary's Hospital,

Manchester University NHS Foundation Trust

Hathersage Road

Manchester

M13 9WL

Dawn Dowding dawn.dowding@manchester.ac.uk

Rohna Kearney rohna.kearney@mft.nhs.uk

Abstract word count: 276

Main article word Count: 2416

Key words: Pessary, self-management, self-care, prolapse, scoping,

For peer review only

What is known from the existing literature about self-management of pessaries for pelvic organ prolapse? A scoping review protocol

Abstract

Introduction

Pelvic organ prolapse (POP) can be managed with a pessary, however, regular follow-up may deter women from pessary management due to the inconvenience of frequent appointments, as well as preventing pessary users from autonomous decision making. Pessary self-management, whereby the woman removes and inserts her own pessary may be a solution to these issues. However, there remains a number of uncertainties regarding the potential benefits and risks of pessary self-management. This scoping review aims to map available evidence about the subject of pessary self-management for POP to identify knowledge gaps providing the basis for future research.

Methods and analysis

The scoping review will be conducted utilising the Joanna Briggs Institute scoping review methodology and reported in accordance with Preferred Reporting Items for Systematic reviews and Meta-Analyses extension for Scoping Reviews PRISMA-ScR guidelines. A search of MEDLINE, CINAHL, EMBASE and PsycInfo will be undertaken to identify relevant articles which meet the eligibility criteria using the search terms 'pessary' and 'self-management' or 'self-care'. A hand search of the reference list of non-original research identified during the search but excluded, will be conducted for additional publications which meet the inclusion and exclusion criteria. Data relevant to the topic of pessary self-management will be extracted and critical appraisal of all included publications undertaken.

Ethics and dissemination

No ethical or Health Research Authority (HRA) approval is required to undertake the scoping review. However, it has been registered with The Open Science Framework (OSF) (DOI 10.17605/OSF.IO/DNGCP). The findings will inform future research exploring pessary self-management and be disseminated via both a presentation at a national conference and publications in peer reviewed journals.

For peer review only

Article Summary

Strengths and limitations of this study

- The scoping review will be undertaken following systematic and rigorous established guidelines to ensure transparency and reproducibility.
- Critical appraisal of the evidence will be undertaken to determine the weighting of included research findings, as well as identifying potential methodological strengths and limitations of the current evidence base.
- The identification and synthesis of data will be limited to published articles found on the MEDLINE, CINAHL, EMBASE and PsycInfo databases and a hand search of reference lists.

For peer review only

Introduction

Pelvic organ prolapse (POP) is defined as the downward displacement of one or more of the pelvic organs including the uterus, vaginal compartments, bowel or bladder [1]. Common POP symptoms reported by women include seeing or feeling a vaginal bulge, a heaviness or a dragging sensation, difficulties voiding or defecating and sexual dysfunction, all of which can significantly negatively impact a woman's quality of life [1].

A pessary is a medical device which can be inserted into the vagina to provide mechanical support to the prolapsed organs. Pessary management offers women with prolapse comparable treatment outcomes to surgery in terms of reported symptoms and quality of life [2] and absence of bulge and no desire for further treatment [3]. This may be a particularly desirable option for women who have not completed their family, are unfit for surgical management or would simply prefer to avoid the risks that POP surgery entails [4]. There are a wide variety of pessaries available, offering a conservative, long-term management option to women. Pessary follow-up is required, tending to be 3-6 monthly [5]. At each appointment the pessary is removed, the vaginal tissues examined and either a new pessary, or the same pessary replaced after cleaning. The need for regular follow-up is often cited as a reason why women opt for surgical management of POP due to the inconvenience of frequent appointments [6, 7, 8]. Moreover, clinician management means women are denied autonomy in how and when to use their pessary. Furthermore, the cost of regular pessary follow-up appointments is not insignificant, with over 86,000 pessaries inserted annually in English NHS services alone [9].

A solution to these frequently cited issues with pessary care may be pessary self-management. This entails the woman removing and reinserting her own pessary as required or desired. There remains a number of uncertainties regarding the benefits and possible risks of pessary self-management [10]. A preliminary search in May 2021 identified a recent review of the published evidence related to pessary self-management, which highlighted current uncertainties within the subject area [10]. These include whether pessary self-management improves a woman's satisfaction, quality of life and therefore long-term use of a pessary; whether pessary self-management is safe and what follow-up requirements are for women self-managing their pessary [10]. However, the review was limited to searching PUBMED and had a focus of patient safety as indicated by the search terms utilised. Therefore, only five eligible studies were identified. A preliminary search of MEDLINE, the Cochrane Database of Systematic Reviews and JBI Evidence Synthesis was conducted and no further systematic or scoping reviews on the topic were identified as being currently underway.

It is acknowledged there is a lack of robust evidence regarding the risks and benefits of pessary self-management due to the small-scale, non-randomised nature of much of the evidence [10]. Whilst there is currently a lack of robust evidence regarding the benefits of self-management, there is qualitative and observational evidence from women using pessaries about the benefits pessary self-management offers to them, including flexibility in how and when they use the pessary [6, 11].

This scoping review aims to map available evidence about the subject of pessary self-management for POP to identify knowledge gaps [12] providing the basis for future research.

For peer review only

The following research question was formulated: What is known from the literature about pessary self-management for women with POP?

Methods and analysis

Types of Sources

The scoping review will be conducted utilising the Joanna Briggs Institute scoping review methodology [13] and reported in accordance with Preferred Reporting Items for Systematic reviews and Meta-Analyses extension for Scoping Reviews PRISMA-ScR guidelines (Supplementary material) [14]. As advocated by PRISMA-ScR guidelines the review has been registered with The Open Science Framework (OSF) (DOI 10.17605/OSF.IO/DNGCP) and the protocol published to ensure transparency and reproducibility.

To be eligible for inclusion in the review, papers must include original research data regarding pessary self-management for women with POP. Pessary self-management and what it entails is poorly defined. However, for the purpose of this scoping review women who remove or insert their pessary independently will be classed as self-managing. There will be no time limit set as it is acknowledged pessary management of prolapse is a long-standing treatment option and therefore self-management of pessaries may also be. All identified studies will be included regardless of the methodology utilised to ensure the different aspects and perspectives of pessary self-management for POP will be explored. Non-original articles such as reviews will be excluded to avoid duplication of evidence. However, the reference list of all review articles identified in the search will be checked and all relevant original research cited will be included within this review.

The search aims to include publications from different methodological perspectives and to scope the available evidence related to pessary self-management for POP without presuming what the important outcomes may be. Therefore, rather than the PICO tool which is typically used for systematic reviews of quantitative evidence, the population, concept and context of the research question will be utilised to structure the search [15].

Population

The population in focus are women with pelvic organ prolapse who use a pessary. It is recognised the prevalence and severity of pelvic organ prolapse increases significantly with increasing age [16]. However, women of all ages develop prolapse, with 77% of 18-29-year-old women examined in a routine gynaecological clinic having stage one or two prolapse [16]. Therefore, no limits will be placed on the age of the study population. Pelvic organ prolapse is typically measured using the POPQ system [17], the measurements of which can be used to determine the stage of prolapse [17]. It is acknowledged the anatomical extent of a prolapse as measured by staging may not correlate with the severity of a woman's symptoms [18]. Therefore, it is advocated women be treated based upon the bothersomeness of their symptoms. It can thus be assumed women managing their

For peer review only

prolapse symptoms with a pessary were sufficiently bothered to consent to treatment, and the stage of their prolapse is of less significance than this. Therefore, the review will not exclude women based upon the stage of their prolapse.

Concept

The concept to be explored is specifically those who self-manage their pessary for pelvic organ prolapse. That included studies must relate to this type of pessary is an important distinction, as pessaries can be for either structural or medicinal purposes [19]. Pharmaceutical pessaries are solid tablets containing medicinal products for insertion into the vagina and are typically prescribed when a local effect is desired [19]. While there is limited evidence supporting the use of vaginal oestrogen as a treatment for pelvic organ prolapse [20], National Institute for Health and Care Excellence (NICE) guidelines suggest clinicians consider the use of local oestrogen cream or pessaries for women with symptomatic prolapse and vaginal atrophy [21]. Therefore, there is potential for articles with a focus of pharmaceutical pessaries for women with pelvic organ prolapse to be identified during the search. The term mechanical pessaries includes devices designed to manage symptoms of urinary incontinence [22], with some devices having a dual purpose for management of prolapse and urinary symptoms [22]. Studies will be excluded if the pessaries are solely for urinary incontinence, however studies with a sample population that includes women using dual purpose pessaries are eligible for inclusion. There is a broad range of pessaries available to manage pelvic organ prolapse and these are typically classed as either a support or space occupying pessary [17]. While the pessaries have different mechanisms of working, both have the same purpose of reducing the descent of, and symptoms associated with, prolapse. Therefore, studies including both types of pessaries will be included.

Context

Whether there are differences in the extent to which pessary self-management is offered to, or accepted by women, depending on the country they live in, healthcare provision, or the culture they belong to, is of particular interest to the authors. Therefore, there is no specific context to the question as the authors are interested in evidence related to pessary self-management for POP regardless of the setting where self-management was initiated, or within what country.

Articles will be excluded if they are not accessible in the English language due to feasibility issues, it is acknowledged this may limit the scope and generalisability of findings. Relevant conference abstracts will be included if the full data set has not subsequently been published. While this prevents the authors from appraising the full dataset and detailed description of the study processes, it enables the inclusion of recent research studies which have not yet been published as well as findings which may not be deemed sufficiently significant by the authors to publish.

The search strategy will aim to locate published studies related to pessary self-management. A search of MEDLINE, CINAHL, EMBASE and PsycInfo will be undertaken to identify articles

For peer review only

on the topic. The search terms which will be used for all databases are pessary and self-management or self-care. Searches of all databases will be undertaken between 5th and 7th of May 2021. Hand searches will be undertaken throughout May 2021. Data extraction, critical appraisal and synthesis of the results will be undertaken following this.

Inclusion criteria

- Original research
- Pessary for POP
- Published in English language
- Focuses on self-management of pessary for POP

Exclusion criteria

- Not relevant to subject area
- Not published in English language
- Not original research including case reports

<u>Study/Source of Evidence selection</u>

Following the search, all identified citations will be collated and uploaded and duplicates and non-original research publications removed. The abstracts will then be reviewed for relevance to the review question. In the instance of abstracts which do not explicitly refer to pessary self-management but are relevant to pessaries for prolapse, the full text will be reviewed to check for references to pessary self-management which may not have been deemed as sufficiently significant to include within the abstract. All data identified which is relevant to pessary self-management will be extracted regardless of the overall aim of the publication, the care setting and the level of focus upon pessary self-management. A sample of 20% of abstracts will be screened by an independent reviewer to ensure agreement with inclusion or exclusion decision. In the instance of disagreement regarding included or excluded studies not resolved through discussion, a third reviewer will be asked to make the final decision. Potentially relevant sources will be retrieved in full and assessed in detail against the inclusion criteria. Reasons for exclusion of sources of evidence that do not meet the inclusion criteria will be recorded and reported in the scoping review.

<u>Data Extraction</u>

Data will be extracted from papers included in the scoping review using a data extraction tool developed by the reviewers based upon scoping review guidance [13]. The extracted data will be entered into the tool electronically via Microsoft Excel. The data extraction form will include details of the author(s), year of publication, population studied, context, methodology utilised and key findings relevant to pessary self-management. A second reviewer will perform data extraction from a subset of 10% of included articles to ensure a standardised process. If there are discrepancies between both extractions, the potential

For peer review only

reasons for this will be explored and the data extraction tool modified accordingly to reduce the likelihood for future discordance. Any amendments to the data extraction tool will be recorded including the reasons for this, for transparency. The final version of the data extraction tool will be included in the scoping review to ensure reproducibility.

Quality appraisal

Appraising the quality of included studies and the subsequent findings is not typically performed during a scoping review [15]. However, for the purpose of this review, quality will be assessed to determine the weighting of included research findings, as well as identifying potential methodological strengths and limitations of the current evidence base. As identified studies will be included regardless of the methodology utilised, it is important to identify a quality appraisal tool which can assess qualitative, quantitative and mixed methods research. Therefore, the updated Mixed Methods Appraisal Tool (MMAT) will be utilised [23]. The MMAT was initially developed in 2006 and has subsequently been revised to facilitate more efficient quality assessment, therefore the most recently developed 2018 MMAT will be used for this review [23]. The authors of the revised MMAT discourage reviewers from calculating a single overall score to determine the methodological quality of a paper as it prohibits readers from understanding the specific strengths and limitations of a study [23] Therefore, the quality appraisal of each included study will be presented in a tabular format (Figure 1), detailing the rating for each criterion assessed using the MMAT. The same subsample of studies randomly selected by the second reviewer for data extraction quality assurance, will be quality appraised by the second reviewer to ensure agreement in the assessment process. The 2018 version of the MMAT has improved content validity compared with previous versions, however the interrater reliability has yet to be established [24]. Therefore, ensuring there is concordance between both reviewer's assessment of quality will be an important step in the scoping review process.

Patient and public involvement

Members of the public and pessary users have not directly been involved with development of this protocol or review process. However, the need for research exploring pessary self-management was highlighted by The James Lind Alliance (JLA) Priority Setting Partnership for pessary and prolapse [25]. Several women with experience of pessaries participated in this partnership either as members of the steering group, by attending the consensus workshop or completing questionnaires. Understanding more about self-management was ranked third out of 20 priorities by the JLA Priority Setting Partnership. The topic of the scoping review has therefore previously been identified and prioritised by patients and members of the public.

Data Analysis and Presentation

The data will be analysed and presented in numerical and tabular format to describe the current evidence base, for example the extent of identified literature, the context of included research such as the country of origin and the nature of the research. This will

For peer review only

provide a scope of the existing evidence related to pessary self-management as a key objective of this review. Furthermore, the key findings of included research will be analysed for emerging themes. A narrative description of the themes and a summary of the evidence related to this will be presented in text and numerical format as appropriate.

Ethics and dissemination

No ethical or Health Research Authority (HRA) approval is required to undertake the scoping review. However, it has been registered with The Open Science Framework (OSF) (DOI 10.17605/OSF.IO/DNGCP) to ensure quality through transparent reporting and to prevent overlapping or duplicate work being undertaken prior to publication of the review findings [26]. The scoping review will be disseminated by publication in a peer reviewed journal and submitted for presentation at a national conference. The findings will also inform subsequent exploratory work regarding pessary self-management.

For peer review only

Authors contributions

Lucy Dwyer devised the scoping review question, methodology and drafted this manuscript.

Rohna Kearney substantively contributed to the development of the scoping review question, methodology and revised and approved this manuscript.

Dawn Dowding substantively contributed to the development of the scoping review question, methodology and revised and approved this manuscript.

Competing interests statement

Lucy Dwyer and Rohna Kearney are co-applicants of the NIHR/HTA funded Treatment of Prolapse with Self-Care Pessary (TOPSY) study.

Funding

Lucy Dwyer, Clinical Doctoral Research Fellow, NIHR300519 is funded by Health Education England (HEE) / National Institute for Health Research (NIHR) for this research project. The views expressed in this publication are those of the author(s) and not necessarily those of the NIHR, NHS or the UK Department of Health and Social Care.

For peer review only

References

1. Haylen, B.T., Maher, C.F., Barber, M.D. *et al*, 2016. An International Urogynecological Association (IUGA) / International Continence Society (ICS) joint report on the terminology for female pelvic organ prolapse (POP). Int Urogynecol J 27, 165–194 (2016). doi.org/10.1007/s00192-015-2932-1
2. Lone F, Thakar R, Sultan AH, 2015 One-year prospective comparison of vaginal pessaries and surgery for pelvic organ prolapse using the validated ICIQ-VS and ICIQ-UI (SF) questionnaires. Int Urogynecol J. 2015 Sep;26(9):1305-12. doi: 10.1007/s00192-015-2686-9. Epub 2015 Apr 11. PMID: 25862241
3. Miceli, A., Dueñas-Diez, JL, 2019. Effectiveness of ring pessaries versus vaginal hysterectomy for advanced pelvic organ prolapse. A cohort study. Int Urogynecol J 30, 2161–2169 (2019). https://doi.org/10.1007/s00192-019-03919-8
4. Kapoor, D.S., Thakar, R., Sultan, A.H. *et al*, 2009. Conservative versus surgical management of prolapse: what dictates patient choice?. Int Urogynecol J 20, 1157 (2009). https://doi.org/10.1007/s00192-009-0930-x
5. Gorti M, Hudelist G, Simons A, 2009. Evaluation of vaginal pessary management: a UK-based survey. J Obstet Gynaecol. 2009 Feb;29(2):129-31. doi: 10.1080/01443610902719813. PMID: 19274547
6. Kearney R, Brown C, 2014. Self-management of vaginal pessaries for pelvic organ prolapse. BMJ Open Quality 2014;3:u206180.w2533. doi: 10.1136/bmjquality.u206180.w2533
7. Basu, M., Wise, B. and Duckett, J., 2011. A qualitative study of women's preferences for treatment of pelvic floor disorders. BJOG: An International Journal of Obstetrics & Gynaecology, 118(3), pp.338-344.
8. Nguyen, J. N. , Jones, C. R. & Krissovich, M, 2005. Pessary Treatment of Pelvic Relaxation. Journal of Wound, Ostomy and Continence Nursing, 32(4), 255–261.
9. Secondary Care Analytical Team, NHS Digital, 2020. Hospital Outpatient Activity 2019-20. Accessed online at digital.nhs.uk/data-and-information/publications/statistical/hospital-outpatient-activity on 10/05/21
10. Bugge C, Dembinsky M, Kearney R, Hagen S, 2021. Does self-management of vaginal pessaries improve care for women with pelvic organ prolapse? BMJ 2021; 372 :n310 doi:10.1136/bmj.n310
11. Storey, S., Aston, M., Price, S., Irving, L. and Hemmens, E, 2009, Women's experiences with vaginal pessary use. Journal of Advanced Nursing, 65: 2350-2357. https://doi.org/10.1111/j.1365-2648.2009.05095.x
12. Munn, Z., Peters, M.D.J., Stern, C. *et al*, 2018. Systematic review or scoping review? Guidance for authors when choosing between a systematic or scoping review approach. BMC Med Res Methodol 18, 143 (2018).
13. Peters. MDJ, Godfrey. C, McInerney. P, Munn. Z, Tricco. AC, Khalil, H, 2020. Chapter 11: Scoping Reviews (2020 version). In: Aromataris E, Munn Z (Editors). JBI Manual for Evidence Synthesis,

14. Tricco AC, Lillie E, Zarin W, O'Brien KK, Colquhoun H, Levac D, *et al*, 2018. PRISMA Extension for Scoping Reviews (PRISMAScR): Checklist and Explanation. Ann Intern Med. 2018;169:467–473
15. Peters, Micah D.J. Godfrey, Christina M. Khalil, Hanan; McInerney, Patricia Parker, Deborah; Soares, Cassia Baldini, 2015.s Guidance for conducting systematic scoping reviews, International Journal of Evidence-Based Healthcare: September 2015 - Volume 13 - Issue 3 - p 141-146 doi: 10.1097/XEB.0000000000000050
16. Swift. S, 2000. The distribution of pelvic organ support in a population of female subjects seen for routine gynecologic health care. American Journal of Obstetrics and Gynecology. Volume: 183 (2) 277-285
17. Dwyer. L and Kearney. R, 2021. Conservative management of pelvic organ prolapse, Obstetrics, Gynaecology & Reproductive Medicine, Volume 31 (2) 35-41
18. Gutman, R. E., Ford, D. E., Quiroz, L. H., Shippey, S. H., & Handa, V. L, 2008. Is there a pelvic organ prolapse threshold that predicts pelvic floor symptoms?. American journal of obstetrics and gynecology, 199(6), 683.e1–683.e6837.
19. Watson, J., Rees, J.A. and Smith, I. eds., 2014. Pharmaceutical practice. Elsevier Health Sciences
20. Ismail SI, Bain C, Hagen S, 2010. Oestrogens for treatment or prevention of pelvic organ prolapse in postmenopausal women. Cochrane Database of Systematic Reviews 2010, Issue 9. Art. No.: CD007063. DOI: 10.1002/14651858.CD007063.pub2. Accessed 08 June 2021.
21. National Institute for Health and Care Excellence, 2019. Urinary incontinence and pelvic organ prolapse in women: management. Accessed online at www.nice.org.uk/guidance/ng123 on 08/06/2021.
22. Amir-Khalkhali, B. and Farell, S.A., 2006. Selection of pessaries for urinary incontinence. In Pessaries in Clinical Practice (pp. 46-53). Springer, London.
23. Hong, Q.N., Fàbregues, S., Bartlett, G. *et al*, 2018. The Mixed Methods Appraisal Tool (MMAT) version 2018 for information professionals and researchers. Education for Information, 34 (4). pp. 285-291. ISSN 0167-8329
24. Hong, Q. N., Pluye, P., Fàbregues, S., Bartlett, G., Boardman, F., Cargo, M., Dagenais, P., Gagnon, M. P., Griffiths, F., Nicolau, B., O'Cathain, A., Rousseau, M. C., & Vedel, I, 2019. Improving the content validity of the mixed methods appraisal tool: a modified e-Delphi study. Journal of clinical epidemiology, 111, 49–59.e1.
25. Lough K, Hagen S, McClurg D the JLA Pessary PSP Steering Group, *et al*, 2018. Shared research priorities for pessary use in women with prolapse: results from a James Lind Alliance Priority Setting Partnership. BMJ Open, April 2018, doi: 10.1136/bmjopen-2017-021276
26. G. M. Tawfik, H. T. N. Giang, S. Ghozy, A. M. Altibi, H. Kandil, H.-H. Le, et al, 2020. Protocol registration issues of systematic review and meta-analysis studies: a survey of global researchers. BMC Medical Research Methodology 2020 Vol. 20 Issue 1 Pages 213. DOI: 10.1186/s12874-020-01094-9

For peer review only

Figure legends

Figure 1- Example of presentation of Mixed Methods Appraisal Tool results

For peer review only

Figure 1

Studies	Criteria from the Mixed Methods Appraisal Tool																								
	1.1	1.2	1.3	1.4	1.5	2.1	2.2	2.3	2.4	2.5	3.1	3.2	3.3	3.4	3.5	4.1	4.2	4.3	4.4	4.5	5.1	5.2	5.3	5.4	5.5
Author, date	0	1	1	1	1											1	0	1	1	1	1	1	1	1	1
Author, date											0	1	1	1	1										
Author, date						1	1	1	0	1															

For peer review only

http://mixedmethodsappraisaltoolpublic.pbworks.com/w/file/fetch/140056890/Reporting%20the%20results%20of%20the%20MMAT.pdf

Preferred Reporting Items for Systematic reviews and Meta-Analyses extension for Scoping Reviews (PRISMA-ScR) Checklist

SECTION	ITEM	PRISMA-ScR CHECKLIST ITEM	REPORTED ON PAGE #
**TITLE**			
Title	1	Identify the report as a scoping review.	1
**ABSTRACT**			
Structured summary	2	Provide a structured summary that includes (as applicable): background, objectives, eligibility criteria, sources of evidence, charting methods, results, and conclusions that relate to the review questions and objectives.	2
**INTRODUCTION**			
Rationale	3	Describe the rationale for the review in the context of what is already known. Explain why the review questions/objectives lend themselves to a scoping review approach.	4
Objectives	4	Provide an explicit statement of the questions and objectives being addressed with reference to their key elements (e.g., population or participants, concepts, and context) or other relevant key elements used to conceptualize the review questions and/or objectives.	4
**METHODS**			
Protocol and registration	5	Indicate whether a review protocol exists; state if and where it can be accessed (e.g., a Web address); and if available, provide registration information, including the registration number.	5
Eligibility criteria	6	Specify characteristics of the sources of evidence used as eligibility criteria (e.g., years considered, language, and publication status), and provide a rationale.	5 and 7
Information sources*	7	Describe all information sources in the search (e.g., databases with dates of coverage and contact with authors to identify additional sources), as well as the date the most recent search was executed.	5
Search	8	Present the full electronic search strategy for at least 1 database, including any limits used, such that it could be repeated.	Included
Selection of sources of evidence†	9	State the process for selecting sources of evidence (i.e., screening and eligibility) included in the scoping review.	7
Data charting process‡	10	Describe the methods of charting data from the included sources of evidence (e.g., calibrated forms or forms that have been tested by the team before their use, and whether data charting was done independently or in duplicate) and any processes for obtaining and confirming data from investigators.	7
Data items	11	List and define all variables for which data were sought and any assumptions and simplifications made.	5-7
Critical appraisal of individual sources of evidence§	12	If done, provide a rationale for conducting a critical appraisal of included sources of evidence; describe the methods used and how this information was used in any data synthesis (if appropriate).	8

For peer review only - http://bmjopen.bmj.com/site/about/guidelines.xhtml

SECTION	ITEM	PRISMA-ScR CHECKLIST ITEM	REPORTED ON PAGE #
Synthesis of results	13	Describe the methods of handling and summarizing the data that were charted.	8
**RESULTS**			
Selection of sources of evidence	14	Give numbers of sources of evidence screened, assessed for eligibility, and included in the review, with reasons for exclusions at each stage, ideally using a flow diagram.	n/a-to be published with results
Characteristics of sources of evidence	15	For each source of evidence, present characteristics for which data were charted and provide the citations.	n/a-to be published with results
Critical appraisal within sources of evidence	16	If done, present data on critical appraisal of included sources of evidence (see item 12).	n/a-to be published with results
Results of individual sources of evidence	17	For each included source of evidence, present the relevant data that were charted that relate to the review questions and objectives.	n/a-to be published with results
Synthesis of results	18	Summarize and/or present the charting results as they relate to the review questions and objectives.	n/a-to be published with results
**DISCUSSION**			
Summary of evidence	19	Summarize the main results (including an overview of concepts, themes, and types of evidence available), link to the review questions and objectives, and consider the relevance to key groups.	n/a-to be published with results
Limitations	20	Discuss the limitations of the scoping review process.	n/a-to be published with results
Conclusions	21	Provide a general interpretation of the results with respect to the review questions and objectives, as well as potential implications and/or next steps.	n/a-to be published with results
**FUNDING**			
Funding	22	Describe sources of funding for the included sources of evidence, as well as sources of funding for the scoping review. Describe the role of the funders of the scoping review.	10

JBI = Joanna Briggs Institute; PRISMA-ScR = Preferred Reporting Items for Systematic reviews and Meta-Analyses extension for Scoping Reviews.

* Where *sources of evidence* (see second footnote) are compiled from, such as bibliographic databases, social media platforms, and Web sites.

† A more inclusive/heterogeneous term used to account for the different types of evidence or data sources (e.g., quantitative and/or qualitative research, expert opinion, and policy documents) that may be eligible in a scoping review as opposed to only studies. This is not to be confused with *information sources* (see first footnote).

‡ The frameworks by Arksey and O'Malley (6) and Levac and colleagues (7) and the JBI guidance (4, 5) refer to the process of data extraction in a scoping review as data charting.

§ The process of systematically examining research evidence to assess its validity, results, and relevance before using it to inform a decision. This term is used for items 12 and 19 instead of "risk of bias" (which is more applicable to systematic reviews of interventions) to include and acknowledge the various sources of evidence that may be used in a scoping review (e.g., quantitative and/or qualitative research, expert opinion, and policy document).

From: Tricco AC, Lillie E, Zarin W, O'Brien KK, Colquhoun H, Levac D, et al. PRISMA Extension for Scoping Reviews (PRISMAScR): Checklist and Explanation. Ann Intern Med. 2018;169:467–473. doi: 10.7326/M18-0850.

St. Michael's
Inspired Care. Inspiring Science.

For peer review only - http://bmjopen.bmj.com/site/about/guidelines.xhtml
